# GERIATRIC PSYCHIATRY OUTPATIENT REACTIONS TO A POST-COVID TRANSITION FROM VIRTUAL TO IN-PERSON CARE

**DOI:** 10.1093/geroni/igae098.3432

**Published:** 2024-12-31

**Authors:** Kathryn Mertz, Samantha Baldinger, Jennifer RIvas, Amir Nikzad, Michael Scarpelli, Blaine Greenwald

**Affiliations:** Zucker Hillside Hospital, Glen Oaks, New York, United States; Zucker Hillside Hospital, Glen Oaks, New York, United States; Zucker Hillside Hospital, Glen Oaks, New York, United States; Zucker Hillside Hospital, Glen Oaks, New York, United States; Zucker Hillside Hospital, Glen Oaks, New York, United States; Zucker Hillside Hospital, Glen Oaks, New York, United States

## Abstract

Because of social distancing/quarantining, the COVID-19 pandemic catalyzed necessary shifts to telepsychiatry in outpatient mental health settings. To enable access and reimbursement for elders, the Centers for Medicaid and Medicare Services (CMS) liberalized restrictive telemedicine regulations. In our Geriatric Psychiatry Clinic, in-person visits quickly shifted to virtual. Tele-video and audio-only services became universally operative between Spring 2020 through May 2023, when – with the public health emergency (PHE) ending - CMS determined hospital-based clinics were ineligible for the ‘facility fee’ if patients were seen virtually, so return to in-person care was strongly encouraged. Shortly thereafter, to determine impact, consecutive geriatric psychiatry clinic patients over a several days period were surveyed re: satisfaction with virtual versus in-person care, and about challenges experienced returning in-person. Of 134 patients surveyed, 43% had engaged in tele-video, 43% in audio-only, and 17% utilized both. Utilizing a 5 -point Likert scale (very unsatisfied = 1 to very satisfied = 5), virtual (video, audio or both) (mean = 4.1) and in-person (mean = 4.2) care did not differ (p-value = 0.82 [Wilcoxon signed-rank test]). Amongst challenges associated with returning to in-person, 75% identified medical issues as very difficult/difficult, 46% financial issues, 25% distance to clinic, 23% transportation concerns, and 16% caregiving responsibilities. Taken together, findings suggest that geriatric psychiatry outpatients are comparably satisfied with virtual and in-person visits, however notable proportions experienced challenges reverting to in-person, especially because of medical and financial issues. As such, continuing advocacy supporting virtual options in psychiatric care of elders is warranted.

